# A Critical Appraisal of the Use and Properties of Nickel–Titanium Dental Alloys

**DOI:** 10.3390/ma14247859

**Published:** 2021-12-18

**Authors:** Petra Močnik, Tadeja Kosec

**Affiliations:** Slovenian National Building and Civil Engineering Institute, Dimičeva 12, 1000 Ljubljana, Slovenia; petra.mocnik@zag.si

**Keywords:** NiTi archwire, tribocorrosion, simulated saliva, Ni release

## Abstract

Nickel–titanium (NiTi) archwires are used in dentistry for orthodontic treatment. NiTi alloys have favourable mechanical characteristics, such as superelasticity and shape memory, and are also known as a corrosion-resistant alloy. In specific cases, an archwire could be attacked by certain types of corrosion or wear degradation, which can cause the leaching of metal ions and a hypersensitive response due to increased concentrations of Ni in the human body. A systematic search of the literature retrieved 102 relevant studies. The review paper focuses on three main fields: (i) electrochemical properties of NiTi wires and the effect of different environments on the properties of NiTi wires (fluoride and low pH); (ii) tribocorrosion, a combination of chemical and mechanical wear of the material, and (iii) the biocompatibility of NiTi alloy and its subsequent effect on the human body. The review showed that corrosion properties are affected by microstructure, pH of saliva and the presence of fluorides. A high variation in published results should be, therefore, interpreted with care. The release of nickel ions was assessed using the same unit, showing that the vast majority of metal ions were released in the first few days of exposure, then a stable, steady state was reached. In tribocorrosion studies, the increased concentrations of Ni ions were reported.

## 1. Introduction

**Figure 1:**
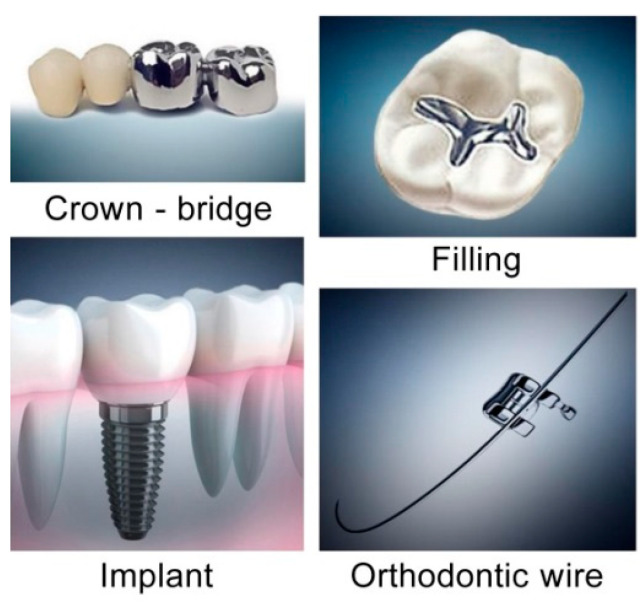
Different metal devices in dentistry.

In dentistry, metals are used for restorative work, dental implants and orthodontic devices [1] (Figure 1). The components used in dental applications are usually made from noble metals (e.g., Au, Pt, Pd, Ag) or highly corrosion-resistant alloys (e.g., Ti alloys and stainless steels). The metals used for such purposes are listed in Table 1.

It is essential that all products are safe for the human body, so no toxic materials should be used for dental applications. Biesiekierski presented the potential hazards of various metallic elements to the human body, detailing the toxicity, carcinogenicity and allergic effect of different elements in Ti alloys [2]. 

The choice of material selected for dental devices depends upon a number of factors including corrosion behaviour, mechanical properties, fabricability, cost, availability, biocompatibility and aesthetic value [6]. In order to achieve the results desired while maintaining absolute safety, implants and other devices intended for biomedical use are regulated globally by various bodies, such as the U.S. Food and Drug Administration (FDA) and the International Organization for Standardization (ISO) [7].

The biocompatibility, i.e., corrosion resistance and release of ions of certain metals and alloys, is maintained by a passive film of surface oxide; the main concern regarding biocompatibility is, therefore, the leakage of metal ions, which happens after the passive film has been damaged [8]. The ion leakage, thus, causes unwanted concentrations of Ni ions in human tissues, which can result in a hypersensitivity response in a certain percentage of patients [1].

NiTi archwire is one of the most common materials used in the 2nd stage of orthodontics treatment. The NiTi alloy is usually composed of about 50% nickel and 50% titanium [9]. The differences in composition are reflected in the properties of the alloy. NiTi alloy provides a constant and controlled force to dental movement and has some special mechanical properties such as superelasticity and shape memory. The biocompatibility of NiTi alloy is, however, still under discussion. It is known that the effect of an aggressive environment is important, especially since titanium and Ti alloys have been found to be sensitive to solutions containing fluoride [10,11,12]. The electrochemical properties of Ti alloys, such as the widely used Ti-6Al-4V alloy, in media containing fluoride have been investigated [12]. It was found that a higher concentration of NaF (≥0.1%) aggravates passive films [12].

Some review papers have been written concerning materials in dentistry, focusing on the properties of titanium [10], the release of metal ions from orthodontic appliances [13,14], nickel–titanium alloys [15], Ti-based shape memory alloys [2], nickel alloys [16], corrosion of certain alloys used in dentistry [6] and, lately, focusing on additive manufacturing methods in the fabrication of NiTi alloys [17]. 

The present literature review focuses on published research concerning the electrochemical properties of NiTi dental alloy archwire and the effect of corrosion upon it. Special emphasis is given to research done in the field of tribocorrosion on NiTi alloy. Research studies exploring the biocompatibility of NiTi archwire are summarized and evaluated.

## 2. Corrosion Properties of NiTi Alloys

NiTi dental archwire is, in its nature, a biomaterial, exposed to a corrosive environment, namely saliva in the human mouth. Biomaterials are, in general, highly corrosion resistant materials, but they are prone to corrosion in certain specific circumstances and when exposed to aggressive corrosive environments. It is, therefore, very important to study the corrosion phenomena of NiTi. There are many studies available that report the corrosion properties of NiTi dental alloys, estimated from electrochemical measurements [18,19,20,21,22,23,24,25,26]. Corrosion test methods are now covered in the lately released standard ISO 10271:20 in the field of dentistry [27] as a result of worldwide demand for standardized test methods for the determination of the acceptability of materials in relation to corrosion. Various solutions are used when studying the properties of biomaterials in body environments in vitro, including a simulated physiological solution, SBF (simulated body fluid) [28], Hank’s solution [21,23,29,30] and solutions with different concentrations of NaCl [31]. To evaluate the electrochemical (corrosion) properties of dental materials, various simulated salivas, such as simulated saliva according to the Fussayama recipe [32,33] or the Duffo recipe [3,4,34,35], have been used. Variations in hygiene and dietary habits can lead to the presence of fluoride, peroxide and/or complex ions in the saliva, which subsequently affect the electrochemical response of the dental alloy. Furthermore, when dental material is exposed to saliva, the pH may change, which can also affect corrosion [32,36,37].

Different types of corrosion on NiTi dental alloys have been reported [21,23,30]. The appearance of crevices [21,30] and pitting corrosion [23] were reported in studies of corrosion of NiTi alloy in simulated saliva. Concerns of galvanic corrosion due to a combination of different materials in the mouth (oral cavity) have been reported in several studies [6,10,11,38]. The presence of a galvanic couple can cause significant corrosion in the material, which can lead to significant elimination of metal ions [10]. 

The present paper focuses and further elaborates on three factors that influence the corrosion behaviour of NiTi material: (i) sample shape and microstructure, (ii) a low pH environment and (iii) the presence of aggressive ions, namely fluoride.

### 2.1. Effect of Production Procedure and Microstructure

Previous research has reported that the microstructure of NiTi alloy significantly impacts its electrochemical properties [4,21]. Longitudinal or cross-sectional pieces may result in different electrochemical behaviour. NiTi dental wires and NiTi sheet material have been electrochemically compared in order to study the effect of the microstructure [4]. Polarization resistances of cross-sections of the wire and the sheet were smaller than polarization resistances of longitudinal surfaces, pointing at greater susceptibility to corrosion of cross-sections [4]. Figueira et al. [21] also studied different sizes (8 and 2 mm diameter) of the NiTi metal samples. From polarization curves and visual examination, they confirmed a greater tendency for crevice corrosion in the case of the smaller sample. 

NiTi wire in the austenitic state is more resistant to corrosion than when in the martensitic state [39]. The same study reports that in dry conditions, plastic deformation predominates in the martensitic state, while fatigue wear predominates in the austenitic state [39]. Another study reports that alloys in the austenitic state had higher breakdown potentials than alloys in the martensitic state [23]. They also claimed that oxide formed on the austenitic phase of NiTi had different chemical properties than oxide formed on the martensitic state [23]. 

**Figure 2:**
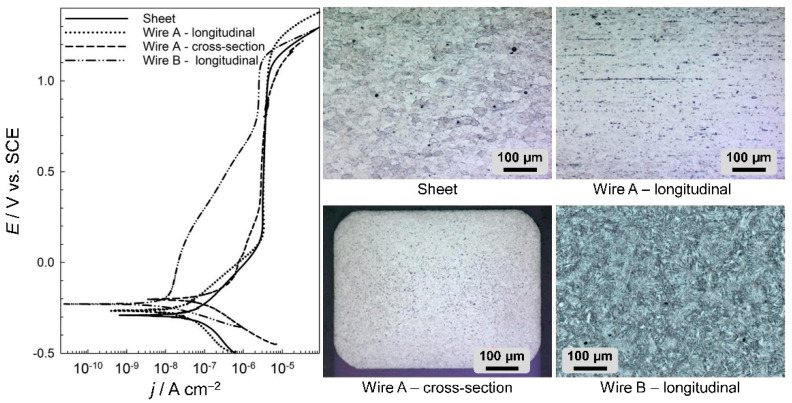
Potentiodynamic curves and representative microstructures of four different NiTi samples: the surface of a 2 mm NiTi sheet, longitudinal and cross-sectional elements of wire A and longitudinal section of NiTi wire B; scan rate: 1 mV/s.

It was also shown that the corrosion resistance varies with the microstructure, which depends on the processing of the material and the direction of observation (longitudinal vs. cross-sectional) [4]. The electrochemical curves and the microstructures of the materials investigated are presented in Figure 2. NiTi dental wires in an artificial saliva solution show passive behaviour [3,40]. The electrochemical properties, as deduced from the presented curves, are presented in Table 2.

Figure 2 shows the potentiodynamic curves (left) and microstructures (right) of various differently shaped metal samples from two types of dental archwire in both the longitudinal and cross-sectional directions and a NiTi sheet. The NiTi samples were as follows: NiTi 3M super elastic archwire, Af = 5–25 °C (denoted as wire A), GAC Dentsply NeoSentalloy archwire, Af = 32.7 °C (denoted as wire B) and a 2-mm NiTi sheet, superelastic, flat annealed, Af = 0 °C (Memry, Gmbh).

The microstructures were analysed using the etching reagents HF, HNO_3_ and H_2_O.

The corrosion current densities, *j*_corr_, of NiTi were higher in the sheet and cross-sectional forms of wire A, at 74.0 and 82.1 nA/cm^2^ respectively, with the comparative values of the longitudinal sections of dental archwires being approximately six times smaller, at 15.5 and 5.41 nA/cm^2^ for wires A and B, respectively. These results confirm the importance of studying the exact shape of any material being investigated, since the microstructure and subsequent electrochemical properties could differ substantially according to the shape of the electrodes—sheet or wire. 

These materials, despite having a similar chemical composition, are manufactured using two completely different technological processes: the wire using rolling and pulling procedures and the sheet produced by hot and cold rolling. Various thermal treatments are carried out during and after these manufacturing processes in order to achieve the desired microstructure and consequent properties of the alloy or product. The microstructures of the samples are shown in Figure 2. The microstructure of the NiTi sheet metal (Figure 2) is martensitic. The average crystal grain size is 30–40 μm (average size G 6.5 according to ASTM standard E112-10 [41], Standard Test Methods for Determining Average Grain Size). Grains are uniformly oriented in all directions due to the hot treatment process. Some non-metallic inclusions are relatively large and have a diameter of about 2–5 µm. The microstructure of the dental wire A (Figure 2) is predominantly martensitic due to the cold deformation process and metallographic examination, whose temperature is lower than the temperature at which phase transformation (martensite to austenite) occurs. Crystal grains are relatively small. The average crystal grain size of the dental wire could not be determined because it was not possible to sufficiently detect all the crystal boundaries by etching. It is noticeable that the crystal grains are slightly elongated in the direction of drawing (Figure 2, wire A—longitudinal). In the cross-section of wire A (Figure 2, wire A—cross-section), there are many inclusions that are more concentrated in the core of the cross-section. The inclusions are stretched longitudinally in the direction of the rolling process, which occurs when producing the dental wire. Computer analysis of the images for wire A showed that inclusions in the cross-section represented 8.8% of the surface area, while, for the sample in the longitudinal direction, inclusions represented, 4.3% of the surface area. A comparison of the size of crystalline grains in wire A and sheet metal revealed a much smaller microstructure in the wire than that in the sheet. The microstructure of the (longitudinal) NiTi wire B predominantly consisted of a fine, needle-like martensite. From the metallographic investigation, it can be concluded that austenite is also present in the microstructure (Figure 2). The inclusions could not be detected or observed.

As evident from the electrochemical results shown in Figure 2 and Table 2, the electrochemical properties of wire B were better than those of wire A when exposed longitudinally (5 cm of wire exposed to saliva). This could be a result of microstructural properties, since larger crystal grains were observed in archwire B. It is also evident that sheet metal has a higher corrosion current density than the longitudinal surface of the archwire. As seen from the results, the cross-section and longitudinal surface of NiTi dental archwire exhibited different electrochemical responses. The differences in electrochemical properties of NiTi alloy are affected by the austenite/martensite transformation temperature, as well as by the procedures during surface finishing by different manufacturers.

It was shown that the composition, shape and microstructure of alloys affect the electrochemical properties of the material investigated and that it is essential to study any particular material in a clearly defined environment. These facts must be taken into account when comparing results in the literature.

### 2.2. Effect of Low pH, Fluoride Concentration and Their Mutual Presence

There are a number of studies of corrosion on dental NiTi wires that focus on the effects of low pH, the presence of fluoride and a combination of both factors [11,26,36,42,43,44,45]. In general, Ti alloys are sensitive to fluoride-containing solutions [10,11,12], resulting in various types of corrosion attack [11,21]. It has been reported that, in the presence of chlorides, the type of corrosion attack is pitting corrosion [26,42]. A low pH also affects the electrochemical properties of Ti alloys [36,46]. Moreover, dental wires are exposed to variations in temperature through cold and hot food and drink, which can also affect their properties [47]. 

Fluoride therapy is recognized as one of the principal methods in the prevention of dental caries [32]. The concentrations of fluorides in various commercial dental hygiene products (ppm in μg/mL, molar and mass concentrations) are presented in Table 3. Table 3 also contains the concentrations of simulated solutions of artificial saliva containing fluoride used in electrochemical studies investigating the properties of NiTi.

**Figure 3:**
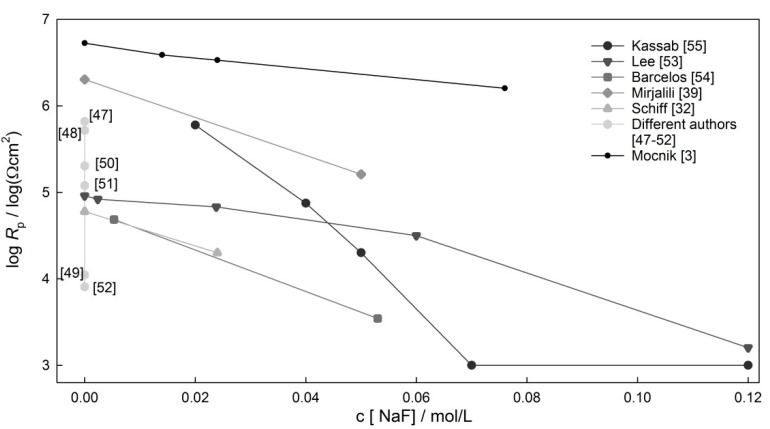
The polarization resistances of NiTi wires in non-acidic media at various fluoride concentrations.

Figure 3 represents the logarithmic values of polarization resistance gathered from data across the literature. *R*_p_ values for NiTi archwires in saliva at a neutral pH were defined at three different concentrations of fluoride: 0.014, 0.024 and 0.076 M [3]. It can be seen from Figure 3 that, in all reported studies, the *R*_p_ values of NiTi alloys decreased as the F concentration increased. Fluoride concentrations higher than 0.076 M NaF are not expected in tooth care products. It can also be seen that very different values of *R*_p_ values are reported under similar conditions (e.g., at NaF concentration of around 0.02 mol/L) [3,32,53]. Furthermore, very different values of *R*_p_ were found at a fluoride concentration of zero, with values varying from 10 kΩ/cm^2^ to 5 MΩ/cm^2^ [3,21,32,40,47,48,49,50,51,52,53].

It is recommended that people wearing NiTi dental appliances limit their use of fluorinated gels [32]. According to research, fluoride is considered to be harmful to the TiO_2_ oxide layer only in acidic environments [44,46]. However, fluoride ions at a neutral pH also have a minor effect on the electrochemical properties [3]. For example, in neutral saliva with a 0.076 M concentration of fluoride ions, a local type of corrosion was indicated by positive hysteresis of the CP curve [3].

When Ti alloy, namely TiO_2_, comes into contact with NaF in an acidic environment, hydrofluoric acid can form causing the Ti oxide layer to dissolve, which results in corrosion [56].

In acidic media, HF dissolves the titanium oxide layer. In the presence of H^+^ ions, NaF can be converted to HF (Reaction (1) [46]). HF is known for its effect on titanium oxide layers (Reactions (2)–(4)) [46].

H^+^ + NaF → Na^+^ + 3 HF (1)

Ti_2_O_3_ + 6 HF → 2 TiF_3_ + 3 H_2_O (2)

TiO_2_ + 4 HF → TiF_4_ + 2 H_2_O (3)

TiO_2_ + 2 HF → TiOF_2_ + H_2_O (4)

When Ti_2_O_3_ dissolves, TiF_3_ is formed (Reaction (2)). When TiO_2_ dissolves (Reactions (3) and (4)), TiF_4_ or TiOF_2_ are formed. Both TiF_3_ and TiOF_2_ are soluble species, meaning that TiO_2_ is no longer protective for the underlying alloy. If the oxide layer is damaged or dissolved, the NiTi alloy is no longer protected, which may result in general or localized corrosion. Soluble nickel chlorides can also form, leading to an increased dissolution and elimination of metal ions. 

The literature has reported some information regarding the stability of TiO_2_ passive film. Huang reported that when NaF was higher than 0.1%, titanium-fluoride containing a complex compound, Na_2_TiF_6_, was formed, thus destroying the TiO_2_ [57]. Nakagawa et al. investigated the limiting conditions for the destruction of a titanium passive layer and found that, for pure Ti, the limit values of pH at which the corrosion resistance of titanium could be maintained were 4.0 and 4.3 in 0.05% NaF and 0.1% NaF, respectively [46].

Oxide films on Ti alloy have better corrosion properties if the alloy contains Ag, Cu, Pd or Pt [58,59]. Specifically, Ti-30Cu-10Ag, Ti-0.5Pd and Ti-0.5Pt alloys promote passivation and, therefore, show better corrosion resistance in the presence of fluoride compared to CP-Ti, Ti-6Al-7Nb and Ti-6Al-4V [58,59,60]. Newly developed Ti alloys combined with one of the elements Mo, Nb, Ta, Zr or Hf have also shown promising biocompatibility and mechanical properties for biomedical applications [60].

A low pH in the oral cavity can result from bacterial activity due to inflammation [61] or be caused by the presence of food or beverages with a low pH. Soft drinks can have a pH of 3.7 (orange juice) right down to pH 2.4 (Coca-Cola). In acidic saliva, NiTi alloy has showed lower resistance to corrosion compared to other dental alloys (TiAl_6_V_4_, pure Ti) [32].

**Figure 4:**
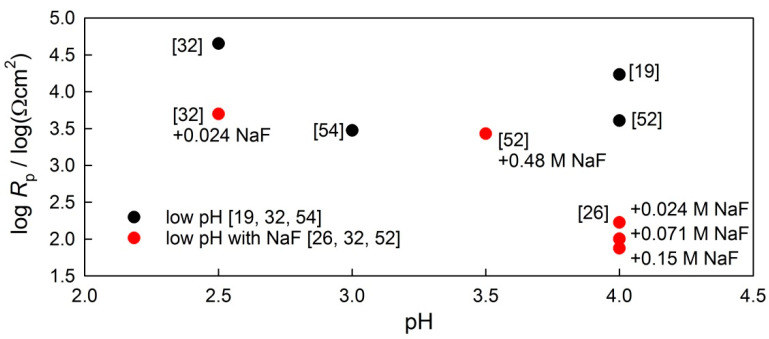
Polarization resistance of NiTi wires in saliva at a low pH with (red dots) and without (black dots) fluoride ions, as gathered from the literature.

Figure 4 represents *R*_p_ values for NiTi archwires in low-pH solutions (from 2.5 to 4), since the pH of the commercially available fluoridated mouthwashes is around 4 and lower. Two types of data are presented, namely low-pH values (black dots) and low-pH values with different concentrations of fluoride (red dots).

In controlled experimental conditions, one would expect the lowering of log *R*_p_ values in line with the lowering of the solution pH, but no clear correlation can be seen regarding the effect of pH. Secondly, at the same pH level, the presence of fluorides leads to a lower *R*_p_ value compared to an environment not containing fluoride. The amount of non-dissociated HF present at different pH values might vary according to the initial concentrations of fluoride at a neutral value.

The effect of low pH, with or without the presence of fluoride, was analysed from the results reported in the literature; large differences in *R*_p_ values were observed in the various experimental conditions reported.

## 3. Tribocorrosion and Wear Properties of NiTi Alloy

**Figure 5:**
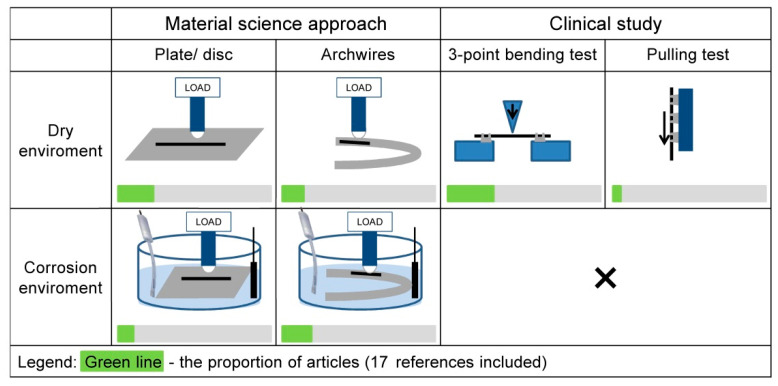
Schematic representation of experimental setups from the literature, which include wear tests or analysis of the mechanical impact of wear [3,4,5,34,39,48,70,71,72,73,74,75,76,77,78,79,80].

Biomaterials in dental applications can degrade by two main mechanisms, namely corrosion and/or mechanical wear. These types of degradation shorten the lifetime of dental materials and, therefore, affect the patient’s quality of life. Furthermore, these degradations can result in metal ion release into the body. Irregularities in the mechanical surface may occur as a result of a number of different things, including the manufacturing process, handling during orthodontic treatment, the mechanical interaction between the archwire and the bracket or the chemical interaction between the archwire and the oral environment. In all cases, such degradation affects the total wear of any given dental material [62]. Loss of material can be even greater when mechanical, chemical or electrochemical processes occur concurrently in a saliva-representing corrosive environment [62]. Most of the published tribocorrosion studies have investigated passive alloys such as stainless steels, titanium alloys or CoCrMo alloy [63,64,65,66,67,68]. In general, these materials are intended for use in a biomedical environment or an environment in which they are exposed to chloride media and/or low-pH environments [69]. Tribocorrosion research on NiTi alloy [4,33,70] is relatively rare; even rarer are tribocorrosion studies on dental wires [3,4,5]. A schematic representation of the different types of study is presented in Figure 5.

In order to appropriately plan the experiment to mimic how the wire is used in clinical practice, one must first be aware of the relative advantages and disadvantages of the experiments planned. Within such experiments, the mechanical and physical properties are defined, which enables appropriate selection of the archwire. Each of the archwire–bracket combinations has a unique set of physical properties that affect its performance [81]. Frictional force in the archwire–bracket slot combination affects tooth movement [75]. To simulate the load of the wire in service with values in range from 0.196 to 0.98 N, the normal load during experiments should be around 1 N [5]. Tooth displacement can be simulated by combining small reciprocating sinusoidal movements and slow linear movements [33]. A low friction coefficient is favoured in orthodontic applications in which lower forces affect dentition in a lower activation period with the desired biological response [5]. The Young’s modulus of NiTi (around 30 GPa) [81] is higher than that of the human bone (around 20 GPa) [82] but is still lower than that of many other alloys (such as stainless steel [2]).

Several tribological experiments have been performed on NiTi alloy in a dry environment [39,71,72,74], with the focus on the wear performances of austenitic and martensitic microstructures [39,71,72]. It has been reported that, in dry tribological conditions, the martensitic state is dominated by plastic deformation and the austenitic state by fatigue wear [39]. A decrease in wear rate at the NiTi–steel contact has been reported due to the cold work hardened surface at the tribological contact [71]. Zhang et al. compared the tribological resistance of NiTi alloy to pure Ti and Ni and found it to be, respectively, 30 times (Ti) and 10 times (Ni) higher [72]. Gialanella et al. reported tribological testing of two different phase NiTi alloys, austenitic and martensitic, with different counter body and loading [73]. The authors concluded that the wear mechanisms depended on a combination of different thermal, mechanical and environmental operating conditions [73]. A study of the effects of friction coefficients on the wear of various dental wires in dry tribological conditions revealed a linear relationship with the hardness of materials, which should be as high as possible for minimum wear [5]. 

In the focused tribological literature in dental applications, friction tests with great and with small (µm) displacement were found [74], in which fretting, i.e., small oscillating displacement, experiments were more related to the clinical environment [74]. Common mechanical tests in orthodontics practice are three-point bending with a distance deflected of 2–3 mm [77,79,80] and pulling tests with a wire–bracket–ligature connection [77]. These are simplified tests that mimic the application of orthodontic archwire.

These tests are done in dry conditions, but in order to estimate the effect of a corrosive environment and mechanical load acting simultaneously, tribocorrosion tests should be conducted.

In the tribocorrosion study of Ti alloy in an oral environment conducted by Golvano et al., the authors summarized that tribocorrosion experiments under OCP (open circuit potential) better resembled in vivo conditions, since tribocorrosion experiments under potentiostatic control can give us additional insights into the underlying tribocorrosion mechanisms [83].

Research into the influence of inhibitors on tribocorrosion of grade 2 Ti in artificial saliva shows that the addition of citric acid or an anodic inhibitor (sodium nitrate) to artificial saliva has little positive effect on material loss (lower wear rate and slightly lower corrosion), due to redox reactions in the contact area [84]. In the presence of a cathodic (calcium carbonate) or organic inhibitor (benzotriazole), significant increases in wear and corrosion rate were observed [84]. In research conducted by Holmes et al., the presence of hard particles (such as alumina) in artificial saliva increased the wear material loss of stainless steel AISI 316L [67]. It is reported that different forms of anodized films affect tribocorrosion behaviour., e.g., rutile in TiO_2_ film significantly decreased mechanical impact during sliding [85]. 

All the studies of tribocorrosion behaviour reported above were conducted using disc samples. Complex shapes and finalized oxide surfaces, such as dental archwires, present a challenge when planning tribocorrosion tests. As a result, to the best of the authors’ knowledge, very scarce or no literature data on tribocorrosion tests on archwires are available up to date. Table 4 and Table 5 summarize different tribological and tribocorrosion setup conditions in the reviewed literature. It can be observed that only a few studies have been conducted on archwires. These tests have been primarily done in a dry environment [81]. Only a few studies were conducted in a corrosive solution [4,5].

It was shown that the total wear of the NiTi sheet increases with an increasing load [4]. Total wear, in general, is as a result of mechanical wear and corrosion process in corrosive solution, such as saliva. Their mutual action can result in higher wear. This synergistic effect depends greatly on the environmental conditions. In the reported study, it was also shown that the electrochemical responses of NiTi sheet and NiTi archwires are very different due to the differences in the microstructure in these two materials.

It is worth pointing out that tribocorrosion properties must be acquired on representative samples in conditions, representative of its use (e.g., archwire in saliva).

## 4. Biocompatibility and Ni Release

Concerns have been raised over NiTi alloy and the fact that nickel is a known allergen that exhibits one of the highest sensitivities in a metallic allergen test [2]. The biocompatibility of biomaterial is related to its corrosion resistance and the degradation of the metal being used [79]. Metal loss adversely affects the biocompatibility of the particular metal [79]; however, no metal or alloy implant is completely inert.

Since high corrosion resistance minimizes the release of Ni ions, it is one of the main prerequisites for good biocompatibility, meaning it is necessary to ensure optimum surface quality for specific applications [87]. Nickel is the most common cause of metal-induced allergic contact dermatitis in humans [88]. It has been reported that 4.5% of the human population has nickel hypersensitivity, with a significantly higher prevalence in females (8% females compared to 0.8% males) [89]. Orthodontic treatment may induce nickel sensitivity [90]. Even if the Ni release does not reach the average dietary intake of nickel, Ni ions may cause a local hypersensitivity reaction at oral soft tissue sites [88]. Nickel-containing orthodontic appliances can, in some cases (e.g., patients with Ni hypersensitivity), cause gingival hyperplasia, labial desquamation, inflammation around lips and swelling and burning sensations affecting the oral mucosa [91,92]. Biocompatibility is a complex field of research that may seem outside the area of interest in orthodontic practice [62].

In general, there are three known methods for investigating the release of metal ions, namely, in vitro, in vivo [93] and a combination of laboratory testing (in vitro testing of in vivo samples). Most studies are conducted under laboratory conditions and report in vitro conditions [91,94,95], but such research cannot simulate bioplastic situations on real samples, so they are sometimes not relevant for clinical use.

There are important clinical, in vivo studies that analysed patients’ saliva and monitored nickel release from orthodontic appliances before and during orthodontic treatment [96,97,98]. The results from research in the years 1997 to 2016 on the concentration of Ni in patients’ saliva in patients with fixed orthodontic treatment were summarized in meta-analysis study [97]. Another study reports the monitoring of nickel release from coated and uncoated NiTI wires after 2 months of orthodontic treatment, in which slightly elevated concentration of Ni were observed in all cases, mostly in the case of uncoated NiTi [99].

In vitro research of the biocompatibility of NiTi with osteoblasts and fibroblasts revealed that NiTi alloy released more Ni into the cell culture media than stainless steel [100]. After two days, however, the concentration of Ni released from both samples stabilized at the same level [100]. Similar findings were reported regarding the release of Ni ions, which increased in the early stages (0–2 weeks), before typically stabilizing due to the formation of a stable oxide film [94,95]. The release of Ni is dependent on the structure of the oxide film. In general, the surface of NiTi will vary across products depending on the preparation process. The main component is stable TiO_2_, which is combined with small amounts of nickel oxides, NiO and Ni_2_O_3_, and metallic Ni [101]. Despite the high proportion of nickel in NiTi alloy, in some studies, Ni-ion release is lower than in the case of other alloys (e.g., stainless steel and Co-Cr-Mo-Ni-Fe alloy) [102]. Another study that reported reduced Ni release focused on reusing and recycling NiTi archwire by using a heat treatment at temperatures of 500 and 600 °C in order to sterilize and potentially restore the original properties. In this study, the Ni release observed in the solution was smaller in the case of the reused wire [103].

Research investigating the 4 week exposure of different archwires (25 samples of stainless steel and 25 samples of nitinol) in artificial saliva reported that the estimated release rates from full-mouth orthodontic appliances are less than 10% of the reported daily dietary intake for nickel [95,104]. Different toxicity levels of Ni ions in the human body are collated in Table 6. Different concentration levels are reported in addition to different measures of contact, for example, whether Ni is consumed via water as an ion or as a Ni alloy in contact with the human skin. It is reported that the release of metals from metallic materials used for biomedical purposes is below the toxicity limit [93] but may lead to allergic reactions [94]. Furthermore, Shabalovskaya reported that the release of Ni ions can occur without any visual signs of corrosion on the NiTi alloy surface [105]. In a review paper focused on the biocompatibility of NiTi alloy in biomedical appliances, authors Es-Souni et al. emphasized the issue of applications involving wear and the low repassivation kinetics of NiTi alloys, which potentially lead to a stronger release of Ni ions [87].

**Table 6:** Toxicity limits of Ni concentrations across different fields of interest.

Limit	Field of Interest	Source	Reference
0.5 µg/(cm^2^·week)	Skin contact	Directive EU, 2004/96/EC, Amending Council Directive 76/769/EEC, as regards restrictions on the marketing and use of nickel for piercing post assemblies for the purpose of adapting its Annex I to technical progress	[106]
7.3 µg/kg body weight	Body burden	Sunderman et al., Biological monitoring of nickel in humans, 1993	[107]
200–300 µg/day	Daily dietary intake	Barret et al., Biodegradation of nickel and chromium in vitro, 1993	[95]
500 µg/day or less	Daily dietary intake	Schroeder et al., Abnormal trace metals in man- nickel, 1962	[104]
2500 ng/L (ppb)	(Inflict) Cell damage	Vreeburg et al., Induction of immunological tolerance by oral administration of nickel and chromium, 1994	[108]
82–406 µg/day/person	From food	WHO 1998 report	[109]
5–25 µg/day/person	From drinking water	WHO 1998 report	[109]
>0.2 µg/L	Healthy serum concentration	Es-Souni et al., Assessing the biocompatibility of NiTi shape memory alloys used for medical applications, 2005	[87]
1–3 µg/L	Healthy urinary excretion	Es-Souni et al., Assessing the biocompatibility of NiTi shape memory alloys used for medical applications, 2005	[87]
0.02 mg/L	Drinking water guideline value	WHO 1998 report	[109]

**Figure 6:**
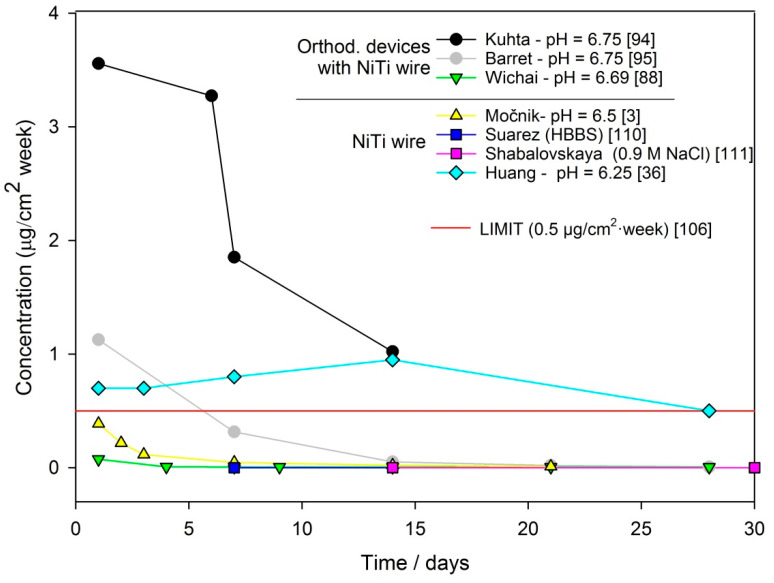
Concentration of Ni ions vs. time of immersion for data reported at pH ~7.

Table 7 summarizes data that have been published from the literature concerning the release of Ni ions from dental archwire or orthodontic devices with NiTi wire during exposure to different artificial saliva. Table 7 contains information (given by authors) on the original values of measured Ni ion concentrations and reported units, the area of exposed surfaces, techniques for analysing the metal ion release and the type and pH of the corrosion media. The reported concentration levels were restated in terms of the mass unit of Ni leached from 1 cm^2^ of material per week, as also reported in the EU document [106]. Recalculation into this unit was carried out in order to be able to compare the reported results. As can be seen from Table 7, experimental setups differ to a great extent between the various studies. The pH values of artificial saliva are between 6 and 7, and in some cases, studies included acidic environments with a pH level around 3.5 [36,39]. The concentration of leached metal ions is higher in more aggressive environments (low pH) and during the early stages of exposure (also seen in Figure 6).

**Table 7:** Ni release from NiTi archwire or orthodontic devices with NiTi wire on exposure to saliva or biological fluid.

Concentration	Unit	Time (Days)	Type of Sample	Technique	Solution/V for Exposure	pH	Author	Recalculated Conc. (µg/cm^2^/week)
11.77	ppb/day	1	Orthodontic devices with NiTi wire	HR-ICP/MS	A. sal. 50 mL	6.75	[94]	3.56
10.83	ppb/day	6						3.27
6.13	ppb/day	7						1.85
3.38	ppb/day	14						1.02
835.1	ppb/day	1				3.5		252
459.5	ppb/day	6						139
138.9	ppb/day	7						42.0
61.9	ppb/day	14						18.7
4.29	ppb	1	Orthodontic devices with NiTi wire	AAS	A. sal. 100 mL	6.75	[95]	1.13
8.41	ppb	7						0.32
2.76	ppb	14						0.052
1.58	ppb	21						0.02
0.70	ppb	28						0.0066
0.62	ppm	1	Orthodontic devices with NiTi wire		A. sal. 25 mL	6.69	[88]	0.075
0.234	ppm	4						0.0071
0.395	ppm	7						0.0068
0.452	ppm	9						0.0061
0.669	ppm	14						0.0058
0.917	ppm	21						0.0053
1.267	ppm	28						0.0055
0.44	µg/L	1	NiTi wire	ICP-MS	A. sal. 100 mL	6.5	[3]	0.39
0.49	µg/L	2						0.22
0.4	µg/L	3						0.12
0.37	µg/L	7						0.047
0.33	µg/L	14						0.021
0.28	µg/L	21						0.012
0.00122	ng/mm^2^	7	NiTi wire	AAS	HBBS	/	[110]	0.000122
0.00160	ng/mm^2^	14						0.00008
0.00204	ng/mm^2^	30						0.00005
Cca. 5	ng/L	14	NiTi wire	ICP-OES	0.9 M NaCl 25 mL	/	[111]	0.000043
Cca. 11	ng/L	30						0.000047
Cca. 20	ng/L	60						0.000043
Cca. 1	µg/cm^2^	1	NiTi wire	AAS	A. sal. 2 mL	3.75	[36]	7.0
Cca. 1	µg/cm^2^	3						2.3
Cca. 2.5	µg/cm^2^	7						2.5
Cca. 3	µg/cm^2^	14						1.5
Cca. 5	µg/cm^2^	28						1.25
Cca. 0.1	µg/cm^2^	1				6.25		0.7
Cca. 0.3	µg/cm^2^	3						0.7
Cca. 0.8	µg/cm^2^	7						0.8
Cca. 1.9	µg/cm^2^	14						0.95
Cca. 2.0	µg/cm^2^	28						0.5

The recalculated concentrations from Table 7 that had been exposed to a neutral pH were plotted into a diagram of concentration vs. time of immersion (Figure 6). In the diagram, the limit of 0.5 µg/cm^2^·week is also outlined, which represents the limit for biocompatibility of Ni alloy in contact with the human skin [106]. Various authors [3,88,94], who have determined the daily release of metal ions from orthodontic archwire, have observed that the most significant releases of metal ions occurred during the first days of exposure. Similarly, the highest Ni ion release from orthodontic devices reported in the first few weeks were in in vivo studies [96,97]. Generally, the concentration of the released Ni ion was below the daily intake level [96,98,99].

In a previous study written by the authors of this review paper, Ni release from new NiTi and stainless steel archwires was monitored during exposure to artificial saliva at 37 °C for 3 weeks [3]. A tribocorrosion experiment was then conducted with a low force and sliding speed in order to mimic the daily use of NiTi and SS orthodontic archwire in saliva. It was found that the Ni concentrations released over one simulated day of use exceeded the limit for safe use of dental archwire. The values of Ni release after simulating 1 day of use was 69.4 μg/cm^2^/week for NiTi wire and 32.6 μg/cm^2^/week for SS wire, which exceeded the limit of 0.5 μg/cm^2^/week by 134 times for NiTi and 65 times for SS [3]. This study showed that the synergistic impact of chemical and mechanical wear is an important factor affecting the total wear of orthodontic archwire, which subsequently affects the release of metal ions.

## 5. Conclusions

This paper reviews state-of-the-art research reported on NiTi dental alloy used as archwires for orthodontic treatment from a material science perspective. Challenges and future perspectives were further discussed. 

Firstly, existing electrochemical data in the published literature were researched and compared. Great differences were revealed in the data, as a result of the corrosive solution used, the shape and microstructure of the samples and the technological finish of the NiTi samples investigated.

Secondly, the effect of low pH and fluoride presence were thoroughly investigated, also showing a high variation in results, which should be interpreted with care.

The third chapter discussed and compared the data published regarding issues surrounding the release of nickel ions. The concentrations released were assembled using the same unit in order to compare the results. It was shown that the vast majority of metal ions released dissolved in the first few days of exposure, then a stable, steady state was reached.

The key part of this review is the overview of the published literature on mechanically influenced corrosion. Very little published data are available, so the tribocorrosion studies can be a powerful tool to predict the performance of NiTi in a complex environment.

In summary, the corrosion and tribocorrosion studies call for a joint effort among various areas of research. 

## Figures and Tables

**Table 1:** Metals used in medical applications, summarized according to [1] with permission from Elsevier *.

Filling	Au Foil; Ag-Sn-(Cu); Amalgam
Inlay, crown, bridge, …	Au-Cu-Ag; Au-Cu-Ag-Pt-Pd; Ti; Ti-6Al-7Nb; Co-Cr; Stainless steels 304 and 316L
Crown—Porcelain to metal	Au-Pt-Pd; Ni-Cr
Solder	Au-Cu-Ag; Au-Pt-Pd
Dental implant	Ti; Ti-6Al-4V; Ti-6Al-7Nb; Au
Orthodontic wire	Stainless steels 304 [3,4] and 316L; Co-Cr; Ti-Ni; Ti-Mo, Ti-Ni-X (X = Co, Cu, …) [5]
Magnetic attachment	Sm-C; Nd-Fe-B; Pt-Fe-Nb; Stainless steels 444, 447J1 and 316L

* Reprinted from *Metals for Biomedical Devices,* 1st Edition, Niinomi, M., *Metals for Biomedical Devices*, Page 7, 2010, with permission from Elsevier.

**Table 2:** Electrochemical parameters of NiTi samples with different microstructures.

Sample	*E*_corr_/V	*j*_corr_ /nA cm^−2^	*E*_b_/V
Sheet (abraded)	–0.291	74.0	1.10
Wire A—longitudinal (abraded)	–0.267	15.5	1.20
Wire A—cross-section (abraded)	–0.200	82.1	1.12
Wire B—longitudinal (as received)	–0.225	5.41	1.13

**Table 3:** Concentration of fluoride ions in products for dental hygiene.

Example of Use	F^–^ (ppm μg/mL)	M(NaF) (mol/L)	Mass % w/w NaF	Studies Using the Same Concentration
Products without fluoride	0	0	0	[3,21,32,40,47,48,49,50,51,52,53]
Mouthwash	100	0.0053	0.022	[54]
	450	0.0237	0.098	[3,32,53]
Children’s toothpaste	500	0.0263	0.109	Not found in reviewed literature
Toothpaste	1000	0.0526	0.217	[40,54,55]
	1450	0.0763	0.315	[3]

**Table 4:** Differences in experimental conditions in studies of dry tribology and tribocorrosion in dental archwires.

Material	Second Body	T (°C)	Media	Load (N)	Sliding Velocity (mm/s)		COF	Author
AISI 304	316L	37	A. sal.	10	Pin-on disc	0.5236 rad/s	0.32	[5]
NiTi	0.56							[5]
TiMo			0.39					[5]
Ti			0.41					[5]
NiTiCu			0.47					[5]
NiTi	Al_2_O_3_	25	A. sal.	1	Reciprocat.	5	0.8	[4]
NiTi	25	A. sal.	2		Reciprocat.	5	0.7	[4]
SS	34	dry	6		0.166	0.187		[81]
Co-Cr	34	dry	6		0.166	0.200		[81]
NiTi	34	dry	6	0.166	0.277			[81]
β-Ti	34	dry	6	0.166	0.467			[81]

**Table 5:** Various mechanical parameters of NiTi alloy derived from dry tribological and tribocorrosion tests.

Type of Sample	Intender Load	Roughness R_a_ (µm)	Vicker’s Hardness	Type of Tribo Experiment	Author
Wire	10 N	0.40	170	Tribocorrosion	[5]
Wire	9.8 N	/	403	Tribocorrosion	[4]
Wire	200 N	0.16	298	Dry	[86]
Wire SE	200 N	0.16	391	Dry	[86]
Wire HE	200 N	0.20	276	Dry	[86]
Wire	9.8 N	0.39	170	Dry	[74]

## Data Availability

Not applicable.

## References

[1] Niinomi M. (2010). Metals for Biomedical Devices.

[2] Biesiekierski A., Wang J., Gepreel M.A.-H., Wen C. (2012). A new look at biomedical Ti-based shape memory alloys. Acta Biomater..

[3] Močnik P., Kosec T., Kovač J., Bizjak M. (2017). The effect of pH, fluoride and tribocorrosion on the surface properties of dental archwires. Mater. Sci. Eng. C.

[4] Kosec T., Močnik P., Legat A. (2014). The tribocorrosion behaviour of NiTi alloy. Appl. Surf. Sci..

[5] Alfonso M.V., Espinar E., Llamas J.M., Rupérez E., Manero J.M., Barrera J.M., Solano E., Gil F.J. (2013). Friction coefficients and wear rates of different orthodontic archwires in artificial saliva. J. Mater. Sci. Mater. Electron..

[6] Upadhyay D., Panchal M.A., Dubey R.S., Srivastava V.K. (2006). Corrosion of alloys used in dentistry: A review. Mater. Sci. Eng. A.

[7] Sidambe A.T. (2014). Biocompatibility of Advanced Manufactured Titanium Implants—A Review. Materials.

[8] Soltis J. (2015). Passivity breakdown, pit initiation and propagation of pits in metallic materials—Review. Corros. Sci..

[9] Kauffman G.B., Mayo I. (1997). The Story of Nitinol: The Serendipitous Discovery of the Memory Metal and Its Applications. Chem. Educ..

[10] Prasad S., Ehrensberger M., Gibson Prasad M., Kim H., Monaco E.A. (2015). Biomaterial properties of titanium in dentistry. J. Oral Biosci..

[11] Schiff N., Boinet M., Morgon L., Lissac M., Dalard F., Grosgogeat B. (2006). Galvanic corrosion between orthodontic wires and brackets in fluoride mouthwashes. Eur. J. Orthod..

[12] Huang H.-H., Lee T.-H. (2005). Electrochemical impedance spectroscopy study of Ti–6Al–4V alloy in artificial saliva with fluoride and/or bovine albumin. Dent. Mater..

[13] Primožič J., Poljšak B., Jamnik P., Kovač V., Jurešić G., Spalj S. (2021). Risk Assessment of Oxidative Stress Induced by Metal Ions Released from Fixed Orthodontic Appliances during Treatment and Indications for Supportive Antioxidant Therapy: A Narrative Review. Antioxidants.

[14] Mikulewicz M., Chojnacka K. (2011). Release of Metal Ions from Orthodontic Appliances by In Vitro Studies: A Systematic Literature Review. Biol. Trace Elem. Res..

[15] Thompson S.A. (2000). An overview of nickel-titanium alloys used in dentistry. Int. Endod. J..

[16] Wataha J.C., Drury J.L., Chung W.O. (2013). Nickel alloys in the oral environment. Expert Rev. Med. Devices.

[17] Yu L., Chen K., Zhang Y., Liu J., Yang L., Shi Y. (2022). Microstructures and mechanical properties of NiTi shape memory alloys fabricated by wire arc additive manufacturing. J. Alloys Compd..

[18] Małkiewicz K., Sztogryn M., Mikulewicz M., Wielgus A., Kaminski J., Wierzchoń T. (2018). Comparative assessment of the corrosion process of orthodontic archwires made of stainless steel, titanium–molybdenum and nickel–titanium alloys. Arch. Civ. Mech. Eng..

[19] Mareci D., Earar K., Zetu I., Bolat G., Crimu C., Istrate B., Munteanu C., Matei M.N. (2015). Comparative electrochemical be-haviour of uncoated and coated niti for dental orthodontic wires. Mater. Plast..

[20] Es-Souni M., Es-Souni M., Fischer-Brandies H. (2002). On the properties of two binary NiTi shape memory alloys. Effects of surface finish on the corrosion behaviour and in vitro biocompatibility. Biomaterials.

[21] Figueira N., Silva T.M., Carmezim M.J., Fernandes J.C.S. (2009). Corrosion behaviour of NiTi alloy. Electrochim. Acta.

[22] Rondelli G. (1996). Corrosion resistance tests on NiTi shape memory alloy. Biomaterials.

[23] Michiardi A., Aparicio C., Planell J.A., Gil F.J. (2007). Electrochemical behaviour of oxidized NiTi shape memory alloys for bio-medical applications. Surf. Coat.Technol..

[24] Trolic I.M., Serdarevic N.L., Todoric Z., Budimir A., Spalj S., Curkovic H.O. (2019). Corrosion of orthodontic archwires in artificial saliva in the presence of Lactobacillus reuteri. Surf. Coat. Technol..

[25] Asserghine A., Medvidović-Kosanović M., Nagy L., Nagy G. (2019). In situ monitoring of the transpassivation and repassivation of the passive film on nitinol biomaterial by scanning electrochemical microscopy. Electrochem. Commun..

[26] Li X., Wang J., Han E.-H., Ke W. (2007). Influence of fluoride and chloride on corrosion behavior of NiTi orthodontic wires. Acta Biomater..

[27] European Committee for Standardization (2020). ISO 10271:2020- Dentistry—Corrosion Test Methods for Metallic Materials.

[28] Liu X.M., Wu S.L., Chu P.K., Chung C.Y., Chu C.L., Yeung K., Lu W.W., Cheung K.M.C., Luk K.D.K. (2007). Effects of water plasma immersion ion implantation on surface electrochemical behavior of NiTi shape memory alloys in simulated body fluids. Appl. Surf. Sci..

[29] Cai W., Sui J.H. (2007). Effect of working pressure on the structure and the electrochemical corrosion behavior of diamond-like carbon (DLC) coatings on the NiTi alloys. Surf. Coat. Technol..

[30] Ng K.W., Man H.C., Yue T.M. (2011). Characterization and corrosion study of NiTi laser surface alloyed with Nb or Co. Appl. Surf. Sci..

[31] Vojtěch D., Fojt J., Joska L., Novák P. (2010). Surface treatment of NiTi shape memory alloy and its influence on corrosion behavior. Surf. Coat. Technol..

[32] Schiff N., Grosgogeat B., Lissac M., Dalard F. (2002). Influence of fluoride content and pH on the corrosion resistance of titanium and its alloys. Biomaterials.

[33] Rapiejko C., Fouvry S., Grosgogeat B., Wendler B. (2009). A representative ex-situ fretting wear investigation of orthodontic arch-wire/bracket contacts. Wear.

[34] Kosec T., Močnik P., Mezeg U., Legat A., Ovsenik M., Jenko M., Grant J.T., Primožič J. (2020). Tribocorrosive Study of New and In Vivo Exposed Nickel Titanium and Stainless Steel Orthodontic Archwires. Coatings.

[35] Duffo G.S., Quezada Castillo E. (2004). Development of artificial saliva solution for studying the corrosion behavior of dental alloys. Corrosion.

[36] Huang H.H., Chiu Y.H., Lee T.H., Wu S.C., Yang H.W., Su K.H., Hsu C.C. (2003). Ion release from NiTi orthodontic wires in artificial saliva with various acidities. Biomaterials.

[37] Huang H.-H. (2005). Surface characterizations and corrosion resistance of nickel-titanium orthodontic archwires in artificial saliva of various degrees of acidity. J. Biomed. Mater. Res. Part A.

[38] Johansson B.I., Bergman B. (1995). Corrosion of titanium and amalgam couples: Effect of fluoride, area size, surface preparation and fabrication procedures. Dent. Mater..

[39] Abedini M., Ghasemi H.M., Ahmadabadi M.N. (2009). Tribological behavior of NiTi alloy in martensitic and austenitic states. Mater. Des..

[40] Mirjalili M., Momeni M., Ebrahimi N., Moayed M.H. (2013). Comparative study on corrosion behaviour of Nitinol and stainless steel orthodontic wires in simulated saliva solution in presence of fluoride ions. Mater. Sci. Eng. C.

[41] ASTM Standard (2010). Standard Test Methods for Determining Average Grain Size.

[42] Ünal H.İ. (2012). Effect of fluoride added artificial saliva solution on orthodontic wires. Prot. Met. Phys. Chem. Surf..

[43] Kocijan A., Merl D.K., Jenko M. (2011). The corrosion behaviour of austenitic and duplex stainless steels in artificial saliva with the addition of fluoride. Corros. Sci..

[44] Sakairi M., Kinjyo M., Kikuchi T. (2011). Repassivation behavior of titanium in artificial saliva investigated with a photon rupture method. Electrochim. Acta.

[45] Chitra P., Prashantha G.S., Rao A. (2020). Effect of fluoride agents on surface characteristics of NiTi wires. An ex vivo investigation. J. Oral Biol. Craniofacial Res..

[46] Nakagawa M., Matsuya S., Shiraishi T., Ohta M. (1999). Effect of fluoride concentration and pH on corrosion behavior of titanium for dental use. J. Dent. Res..

[47] Pakshir M., Bagheri T., Kazemi M.R. (2013). In vitro evaluation of the electrochemical behaviour of stainless steel and Ni-Ti ortho-dontic archwires at different temperatures. Eur. J. Orthod..

[48] Katić V., Otomačić Ćurković H., Semenski D., Baršić G., Marušić K., Špalj S. (2014). Influence of surface layer on mechanical and corrosion properties of nickel-titanium orthodontic wires. Angle Orthod..

[49] Briceño J., Romeu A., Espinar E., Llamas J.M., Gil F.J. (2013). Influence of the microstructure on electrochemical corrosion and nickel release in NiTi orthodontic archwires. Mater. Sci. Eng. C.

[50] Rondelli G., Vicentini B. (1999). Localized corrosion behaviour in simulated human body fluids of commercial Ni-Ti orthodontic wires. Biomaterials.

[51] Schiff N., Grosgogeat B., Lissac M., Dalard F. (2004). Influence of fluoridated mouthwashes on corrosion resistance of orthodontics wires. Biomaterials.

[52] Kao C.-T., Huang T.-H. (2010). Variations in surface characteristics and corrosion behaviour of metal brackets and wires in different electrolyte solutions. Eur. J. Orthod..

[53] Lee T.-H., Huang T.-K., Lin S.-Y., Chen L.-K., Chou M.-Y., Huang H.-H. (2010). Corrosion Resistance of Different Nickel-Titanium Archwires in Acidic Fluoride-containing Artificial Saliva. Angle Orthod..

[54] Barcelos A.M., Luna A.S., Ferreira N.A., Braga A.V.C., do Lago D.C.B., De Senna L. (2013). Corrosion evaluation of orthodontic wires in artificial saliva solutions by using response surface methodology. Mater. Res..

[55] Kassab J.E., Gomes J.P. (2013). Assessment of nickel titanium and beta titanium corrosion resistance behaviour in fluoride and chloride environments. Angle Orthod..

[56] De Castro S.M., Ponces M.J., Lopes J.D., Vasconcelos M., Pollmann M.C.F. (2015). Orthodontic wires and its corrosion—The specific case of stainless steel and beta-titanium. J. Dent. Sci..

[57] Huang H.H. (2003). Effect of fluoride and albumin concentration on the corrosion behavior of Ti–6Al–4V alloy. Biomaterials.

[58] Nakagawa M., Matono Y., Matsuya S., Udoh K., Ishikawa K. (2005). The effect of Pt and Pd alloying additions on the corrosion behavior of titanium in fluoride-containing environments. Biomaterials.

[59] Al-Mayouf A.M., Al-Swayih A.A., Al-Mobarak A., Al-Jabab A.S. (2004). The effect of fluoride on the electrochemical behaviour of Ti and some of its alloys for dental applications. Mater. Corros..

[60] Rodrigues A.V., Oliveira N.T.C., Dos Santos M.L., Guastaldi A.C. (2015). Electrochemical behaviour and corrosion resistance of Ti-15Mo alloy in naturally-aerated solutions, containing chloride and fluoride ions. J. Mater. Sci..

[61] Vasilescu E., Drob P., Ivanescu S., Dan I., Vasilescu C. (2009). Electrochemical behaviour of a new dental alloy for restorative works in simulating extreme functional conditions. Rev. Chim..

[62] Daems J., Celis J.P., Willems G. (2009). Morphological characterization of as-received and in vivo orthodontic stainless steel arch-wires. Eur. J. Orthod..

[63] Mathew M.T., Ariza E., Rocha L.A., Fernandes A.C., Vaz F. (2008). TiCxOy thin films for decorative applications: Tribocorrosion mechanisms and synergism. Tribol. Int..

[64] Mathew M.T., Ariza E., Rocha L.A., Vaz F., Fernandes A.C., Stack M.M. (2010). Tribocorrosion behaviour of TiCxOy thin films in bio-fluids. Electrochim. Acta.

[65] Benea L., Ponthiaux P., Wenger F., Galland J., Hertz D., Malo J.Y. (2004). Tribocorrosion of stellite 6 in sulphuric acid medium: Electrochemical behaviour and wear. Wear.

[66] Bidiville A., Favero M., Stadelmann P., Mischler S. (2007). Effect of surface chemistry on the mechanical response of metals in sliding tribocorrosion systems. Wear.

[67] Holmes D., Sharifi S., Stack M.M. (2014). Tribo-corrosion of steel in artificial saliva. Tribol. Int..

[68] Sun Y., Rana V. (2011). Tribocorrosion behaviour of AISI 304 stainless steel in 0.5M NaCl solution. Mater. Chem. Phys..

[69] Landolt D., Mischler S. (2011). Tribocorrosion of Passive Metals and Coatings.

[70] Tan L., Dodd R.A., Crone W.C. (2003). Corrosion and wear-corrosion behavior of NiTi modified by plasma source ion implantation. Biomaterials.

[71] Abedini M., Ghasemi H.M., Ahmadabadi N.N. (2010). Tribological behaviour of NiTi alloy against 52100 steel and WC at elevated temperatures. Mater. Chem. Phys..

[72] Zhang C., Farhat Z.N. (2009). Sliding wear of superelastic TiNi alloy. Wear.

[73] Gialanella S., Ischia G., Straffelini G. (2008). Phase composition and wear behaviour of NiTi alloys. J. Mater. Sci..

[74] Grosgogeat B., Jablonska E., Vernet J.-M., Jaffrezic N., Lissac M., Ponsonnet L. (2006). Tribological response of sterilized and un-sterilized orthodontic wires. Mater. Sci. Eng. C.

[75] Yu J.H., Huang H.L., Wu L.C., Hsu J.T., Chang Y.Y., Huang H.H., Tsai M.T. (2011). Friction of stainless steel, nickel-titanium alloy and beta-titanium alloy archwires in two commonly used orthodontic brackets. J. Mech. Med. Biol..

[76] Mezeg U., Primožič J. (2017). Influence of long-term in vivo exposure, debris accumulation and archwire material on friction force among different types of brackets and archwires couples. Eur. J. Orthod..

[77] Fernandes F.M.B., Cruz J.M., Magalhães R.C.A. (2015). Comparative Study of NiTi Orthodontic Wires. Mater. Today Proc..

[78] Arici N., Akdeniz B.S., Oz A.A., Gencer Y., Tarakci M., Arici S. (2021). Effectiveness of medical coating materials in decreasing friction between orthodontic brackets and archwires. Korean J. Orthod..

[79] Liu J.K., Lee T.M., Liu I.H. (2011). Effect of loading force on the dissolution behavior and surface properties of nickel-titanium or-thodontic archwires in artificial saliva. Am. J. Orthod. Dentofacial. Orthop..

[80] Lombardo L., Toni G., Mazzanti V., Mollica F., Spedicato G.A., Siciliani G. (2019). The mechanical behavior of as received and retrieved nickel titanium orthodontic archwires. Prog. Orthod..

[81] Kusy R.P., Whitley J.Q. (1990). Effects of surface roughness on the coefficients of friction in model orthodontic systems. J. Biomech..

[82] Wikipedia, Young’s Modulus, Table. https://sl.wikipedia.org/wiki/Pro%C5%BEnostni_modul.

[83] Golvano I., Garcia I., Conde A., Tato W., Aginagalde A. (2015). Influence of fluoride content and pH on corrosion and tribocorrosion behaviour of Ti13Nb13Zr alloy in oral environment. J. Mech. Behav. Biomed. Mater..

[84] Vieira A.C., Ribeiro A.R., Rocha L.A., Celis J.P. (2006). Influence of pH and corrosion inhibitors on the tribocorrosion of titanium in artificial saliva. Wear.

[85] Alves A.C., Oliveira F., Wenger F., Ponthiaux P., Celis J.-P., Rocha L.A. (2013). Tribocorrosion behaviour of anodic treated titanium surfaces intended for dental implants. J. Phys. D Appl. Phys..

[86] Zetu I.N., Romanec C., Mocanu R.M., Cozma C., Ogodescu A. (2013). Physical and mechanical properties of 5 types of orthodontic archwire. Met. Int..

[87] Es-Souni M., Es-Souni M., Fischer-Brandies H. (2005). Assessing the biocompatibility of NiTi shape memory alloys used for medical applications. Anal. Bioanal. Chem..

[88] Wichai W., Anuwongnukroh N., Dechkunakorn S. (2014). Comparison of Chemical Properties and Ni Release of Stainless Steel and Nickel Titanium Wires. Adv. Mater. Res..

[89] Peltonen L. (1979). Nickel sensitivity in the general population. Contact Dermat..

[90] Bass J.K., Fine H., Cisneros G.J. (1993). Nickel hypersensitivity in the orthodontic patient. Am. J. Orthod. Dentofac. Orthop..

[91] Toker S.M., Canadinc D. (2014). Evaluation of the biocompatibility of NiTi dental wires: A comparison of laboratory experiments and clinical conditions. Mater. Sci. Eng. C.

[92] House K., Sernetz F., Dymock D., Sandy J.R., Irelande A.J. (2008). Corrosion of orthodontic appliances—should we care?. Am. J. Orthod. Dentofac. Orthop..

[93] Mikulewicz M., Wołowiec P., Janeczek M., Gedrange T., Chojnacka K. (2014). The release of metal ions from orthodontic appliancesAnimal tests. Angle Orthod..

[94] Kuhta M., Pavlin D., Slaj M., Varga S., Lapter-Varga M., Slaj M. (2009). Type of Archwire and Level of Acidity: Effects on the Release of Metal Ions from Orthodontic Appliances. Angle Orthod..

[95] Barrett R.D., Bishara S.E., Quinn J.K. (1993). Biodegradation of orthodontic appliances. Part I. Biodegradation of nickel and chro-mium in vitro. Am. J. Orthod. Dentofacial. Orthop..

[96] Gölz L., Knickenberg A.K., Keilig L., Reimann S., Papageorgiou S.N., Jäger A., Bourauel C. (2016). Nickel ion concentrations in the saliva of patients treated with self-ligating fixed appliances: A prospective cohort study. J. Orofac. Orthop..

[97] Imani M.M., Mozaffari H.R., Ramezani M., Sadeghi M. (2019). Effect of fixed orthodontic treatment on salivary nickel and chro-mium levels: A systematic review and meta-analysis of observational studies. Dent. J..

[98] Quadras D.D., Nayak U.S.K., Kumari N.S., Priyadarshini H.R., Gowda S., Fernandes B. (2019). In vivo study on the release of nickel, chromium, and zinc in saliva and serum from patients treated with fixed orthodontic appliances. Dent. Res. J..

[99] Masjedi M.K., Niknam O., Jahromi N.H., Javidi P., Rakhshan V. (2016). Effects of Fixed Orthodontic Treatment Using Conventional, Copper-Included, and Epoxy-Coated Nickel-Titanium Archwires on Salivary Nickel Levels: A Double-Blind Randomized Clinical Trial. Biol. Trace Elem. Res..

[100] Ryhänen J., Niemi E., Serlo W., Niemelä E., Sandvik P., Pernu H., Salo T. (1997). Biocompatibility of nickel-titanium shape memory metal and its corrosion behavior in human cell cultures. J. Biomed. Mater. Res..

[101] Okazaki Y., Gotoh E. (2008). Metal release from stainless steel, Co-Cr-Mo-Ni-Fe and Ni-Ti alloys in vascular implants. Corros. Sci..

[102] Ryhänen J. (2000). Biocompatibility of nitinol. Minim. Invasive Ther. Allied Technol..

[103] Gil F.J., Espinar E Llamas J.M., Manero J.M., Ginebra M.P. (2012). Variation of the superelastic properties and nickel release from original and reused NiTi orthodontic archwires. J. Mech. Behav. Biomed. Mat..

[104] Schroeder H.A., Balassa J.J., Tipton I.C.H. (1962). Abnormal trace metals in man-nickel. J. Chronic Dis..

[105] Shabalovskaya S.A. (2002). Surface, corrosion and biocompatibility aspects of Nitinol as an implant material. Bio-Med. Mater. Eng..

[106] European Union (2004). Amending Council Directive 76/769/EEC as Regards Restrictions on the Marketing and Use of Nickel for Piercing Post Assemblies for the Purpose of Adapting its Annex I to Technical Progress. Directive EU 2004/96/EC.

[107] Sunderman F.W. (1993). Biological monitoring of nickel in humans. Scand. J. Work. Environ. Health.

[108] Vreeburg K.J.J., De Groot K., Von Blomberg M., Scheper R.J. (1984). Induction of Immunological Tolerance by Oral Administration of Nickel and Chromium. J. Dent. Res..

[109] WHO (1998). The World Health Report 1998. Life in the 21st Century: A Vision for All.

[110] Suarez C., Vilar T., Gil J., Sevilla P. (2010). In vitro evaluation of surface topographic changes and nickel release of lingual ortho-dontic archwires. J. Mater. Sci. Mater. Med..

[111] Shabalovskaya S.A., Tian H., Anderegg J.W., Schryvers D.U., Carroll W.U., Van Humbeeck J. (2009). The influence of surface oxides on the distribution and release of nickel from Nitinol wires. Biomaterials.

